# Defining the normal appearance of the temporomandibular joints by magnetic resonance imaging with contrast: a comparative study of children with and without juvenile idiopathic arthritis

**DOI:** 10.1186/s12969-018-0223-3

**Published:** 2018-01-24

**Authors:** Matthew L. Stoll, Saurabh Guleria, Melissa L. Mannion, Daniel W. Young, Stuart A. Royal, Randy Q. Cron, Yoginder N. Vaid

**Affiliations:** 1 Department of Pediatrics, University of Alabama at Birmingham, CPP N G10 / 1600 7th Avenue South, Birmingham, AL 35233 USA; 2 Department of Radiology at Children’s of Alabama, CH 2 FL / 1600 7th Avenue South, Birmingham, AL 35233 USA; 3 Present affiliation: Austin Radiological Associates, Austin, TX USA

**Keywords:** Juvenile idiopathic arthritis, Temporomandibular joint, Magnetic resonance imaging

## Abstract

**Background:**

Up to 80% of children with juvenile idiopathic arthritis (JIA) develop arthritis involving their temporomandibular joint (TMJ). Recent studies have questioned the sensitivity of an abnormal MRI in the diagnosis of active arthritis.

**Methods:**

122 children without arthritis undergoing contrast MRI of the head were prospectively consented to undergo a simultaneous contrast MRI of their TMJs. As a comparison point, the initial MRI of the TMJ of 35 newly diagnosed children with JIA were retrospectively scored. The presence and size of effusion and contrast enhancement were measured in the left TMJ in all subjects.

**Results:**

62/122 (51%) controls compared to only 10/35 JIA (29%) patients had an effusion (*p* = 0.022). Contrast enhancement was present in ≥97% of both groups, although the size of the enhancement was, on average, 0.2 mm larger in controls (1.1 ± 0.24 vs 0.88 ± 0.27 mm, *p* <  0.001). Among JIA patients, the size of the enhancement correlated inversely with disease duration (*r* = − 0.475, *p* = 0.005). Chronic changes were present in none of the controls versus 2/35 (5.5%) of the JIA patients (*p* = 0.049).

**Conclusion:**

Findings consistent with minimally active TMJ arthritis appear to be equally likely in children with JIA as compared to non-inflamed controls, while this and other studies confirm that chronic changes are specific to JIA. Thus, small amounts of effusion or contrast enhancement, in the absence of chronic changes, should be interpreted with caution.

**Electronic supplementary material:**

The online version of this article (10.1186/s12969-018-0223-3) contains supplementary material, which is available to authorized users.

## Background

Juvenile idiopathic arthritis (JIA) is the most common form of chronic arthritis in children, affecting approximately 1 in 1000 children [1]. Forty to 80% of children with JIA develop arthritis of the temporomandibular joint (TMJ) [2–4]. TMJ arthritis can occur even in the absence of typical symptoms of pain, stiffness, or swelling, and in the presence of a normal physical exam [2, 5]. Thus, diagnosis is typically made with imaging studies. As radiographs only capture late stages of TMJ arthritis, advanced imaging modalities are typically used, with several studies indicating that magnetic resonance imaging (MRI) may have improved ability to detect TMJ arthritis as compared to ultrasound [5, 6].

A limitation of studies evaluating the use of advanced imaging tools for the diagnosis of TMJ arthritis is the absence of a gold standard, as it would clearly be unethical to subject children to biopsy in order to test the performance characteristics of MRI. In particular, in light of recent evidence indicating that local therapy of TMJ arthritis may impair TMJ growth in children [7], interest in the specificity of an abnormal TMJ MRI has arisen. A retrospective study by Tzaribachev et al. [8] indicated that abnormal MRIs of the pediatric TMJ are rare in those without arthritis elsewhere, with effusions observed in only 3 of 96 children TMJs and enhancement observed in another three children. In contrast, von Kalle et al. [9] retrospectively evaluated the TMJs of 46 children who underwent MRI of the head, finding contrast enhancement in all TMJs with a mean signal intensity of 75% higher than pre-contrast [9]. A subsequent study from the group showed similar contrast signal intensity between patients and controls, with the major difference being in the extent of synovial hypertrophy [10]. Likewise, Ma et al. [11] found that the signal to noise ratio of 24 healthy controls was higher post-contrast as compared to pre-contrast. A similar approach was taken by Resnick et al. [12], who retrospectively evaluated the TMJs of 72 children with JIA and 71 non-inflamed controls. They calculated the enhancement ratio (ER), defined as the intensity of contrast enhancement in the superior TMJ space divided by that of a nearby muscle. They reported significantly higher ER in the JIA patients (2.52 ± 0.79) versus the controls (1.28 ± 0.16), with ROC analysis showing that a cutoff of 1.55 was optimal for distinguishing the two groups. Thus, MRI findings of synovial fluid or mild synovial enhancement in the TMJ may be within normal limits.

A limitation of these studies is that they were retrospective in design, and they were limited to children who underwent contrast MRI of the brain, in whom the TMJ was visualized after the fact. This limitation is potentially problematic, due to lack of use of TMJ coils and other technical issues that may restrict visualization of the joint, although the Resnick study was limited to children with head coils and appropriate visualization of both the TMJ and the surrounding musculature [12]. While the ideal study would be a prospective study of healthy children, such a study would be ethnically challenging due to the requirement for contrast as well as the need for sedation in many young children. Kottke et al. [13] performed a prospective study of children undergoing MRI of the head to evaluate for intracranial pathology, finding joint fluid and contrast enhancement in 83% and 97% of TMJs, respectively, among 27 children studied. To avoid both the limitations of retrospective studies as well as the risks of exposing healthy children to unnecessary contrast MRI studies, we conducted a prospective study of children who underwent contrast MRI of the brain for diagnostic purposes, who agreed to allow us to obtain an MRI of the TMJs simultaneously. We also compared our findings in these controls to findings in children with newly diagnosed JIA undergoing initial MRI screening of the TMJ for arthritis.

## Methods

### Study design

This was a study of children undergoing MRI of the brain/TMJ. Two groups of subjects were included in this study: controls undergoing MRI of the brain were studied prospectively, and children with newly diagnosed JIA undergoing routine MRI of the TMJ were studied retrospectively. This study was approved by the local IRB. Informed consent was obtained from the legal guardians of all individual participants included in the prospective study, as well as participants age 14 or older. Waiver of informed consent was obtained for the retrospective component to the study, as the MRIs of the TMJ were obtained as per standard of care.

### Controls

Children age 1–18 who were undergoing MRI of the brain as per standard of care were considered for the study. Exclusion criteria included (1) active infections; (2) immunodeficiency; (3) known rheumatic disease; (4) sickle cell disease; (5) radiation to the TMJ; (6) TMJ pain; (7) failure to receive contrast enhancement; and (8) excessive motion artifact precluding interpretation of the images. To assess for undiagnosed rheumatic disease, subjects were screened via questionnaire (Additional file 1); anyone who answered affirmatively to any of the questions was excluded.

### Cases

Children age 1–18 newly diagnosed with JIA (symptom onset under 16 years of age) who underwent initial MRI of the TMJ during the same time period during which controls were recruited were identified retrospectively. Their MRIs were reviewed by the same radiologists who reviewed the control studies. Clinical and demographic data were abstracted from their electronic medical records. Maximal incisal opening (MIO) was routinely measured using the Therabite Measuring Scale (Atos Medical, Hörby, Sweden).

### MRI sequences

For the controls, two additional study sequences were obtained in addition to the clinically indicated MRI. All MRIs were performed with an Ingenia 1.5 Tesla (T), Ingenia 3.0 T, or an Avanto 1.5 T scanner (Philips Medical Systems, and Seimens Healthcare, respectively). Both sequences were obtained after all of the clinically indicated sequences and were therefore both post-contrast. These were a fast spin echo T2 and a spin echo T1-weighted fat-saturated sequence of the left TMJ in the sagittal plane. In-plane resolution (pixel size) varied from 0.5–0.8, and slice thickness was 2 mm with no gap.

MRIs of the patients were performed as previously described [14]. For this study, the only images that were retrospectively reviewed were T2-weighted fat-saturated pre-contrast and T1-weighted post-contrast sagittal images of the left TMJ.

### Interpretation

**Fig. 1:**
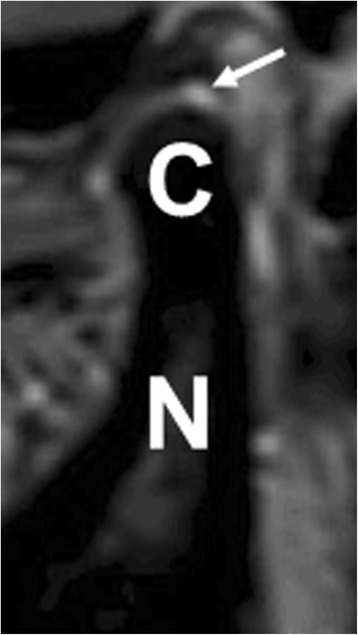
Joint fluid in non-arthritic patient. Sagittal T2W oblique image of the left TMJ in a 14-year-old male subject shows a small amount of joint fluid in the inferior recess measuring 1.2 mm in thickness (arrow). The mandibular condylar head (C) and neck (N) are shown

**Fig. 2:**
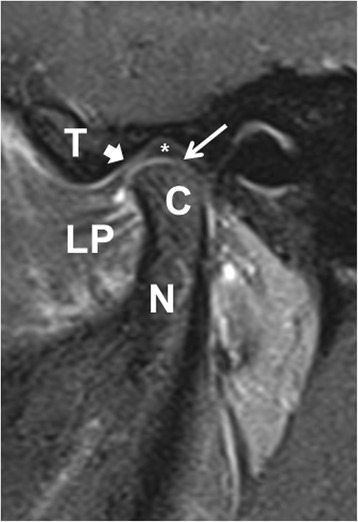
Joint enhancement in non-arthritic patient**.** Post-contrast sagittal T1W oblique image of the left TMJ in a 16-year-old male subject shows enhancement of the synovium in both the inferior (arrow) and superior (arrowhead) recesses with maximal thickness measuring 1.2 mm in thickness. The condylar head (C), condylar neck (N), left lateral pterygoid muscle (LP), glenoid tubercle (T), and meniscus (*) are shown

Interpretation of the MRIs was performed by two board certified pediatric radiologists; one has s 26 years of experience post-training, and the other has 10 years of experience; additionally, the Radiology Department at our hospital interprets approximately 500 pediatric MRIs of the TMJ annually. In both controls and newly diagnosed JIA cohorts, the left TMJ was assessed for the presence or absence of joint fluid (Fig. 1) and synovial enhancement (Fig. 2). Any amount of fluid or synovial enhancement was measured in maximal thickness (in mm), regardless of location (anterior/posterior, inferior/superior joint recesses). The thickness of enhanced synovial lining was measured taking particular care to exclude diffusion of contrast within joint fluid. In addition, chronic changes (pannus formation, condylar flattening, bony erosions, disc deformities, and bony destruction) were assessed in all subjects with the pre-contrast images [14].

### Statistical analysis

Continuous data are presented as means (± SD), and dichotomous data are presented as proportions. The concordance correlation coefficient (CCC) was calculated to quantify the agreement between raters for both the effusion measurement and the enhancement measurement. The CCC measures the degree that matched pairs fall on the concordance line and thus contains the measurements of accuracy and precision. While Cohen’s kappa is often reported as a measure of inter-rater reliability, it is optimally used for categorical or ordinal variables and is less appropriate than the CCC in this case. The CCC was developed as a measure of agreement between continuous variables in a dependent sample [15]. Comparisons between JIA patients and controls, as well as between 1.5 T versus 3 T MRI scanners, were performed with the Chi squared or Fisher exact tests as appropriate for proportional data, and the Student’s t-test for continuous data. The Pearson correlation coefficient was used to assess correlations between continuous variables. The CCC calculation was performed using SAS software, Version 9.3 (copyright, SAS Institute, Inc., Cary, NC, USA).

### Ethical approval

All procedures performed in studies involving human participants were in accordance with the ethical standards of the institutional and/or national research committee, and with the 1964 Helsinki declaration and its later amendments or comparable ethical standards.

## Results

### Subjects

One-hundred-and-twenty-two arthritis-free control subjects were included in the study. Their demographic features and clinical indications for MRI are summarized in Table 1; 38 (31%) had a history of malignancy, of whom 12 (9.8% of the total population) had received chemotherapy or radiation; the rest of the children with malignancy were presumably enrolled at or near the time of diagnosis. None of the subjects had any historical features suggestive of arthritis, nor had any been evaluated by pediatric rheumatology. As a comparison, 35 children with JIA were included; their demographic and clinical information is summarized in Table 2. Of the 17 children using biologic therapies, 14 were taking tumor necrosis factor inhibitors, and 3 (all with systemic JIA) were on the interleukin-1 receptor antagonist, anakinra. The duration from diagnosis to MRI ranged from one day to just under six months, with a mean of 2.2 ± 1.7 months.

**Table 1:** Demographic and clinical features of the control population

Feature	Number
n	122
Sex	
Male	67 (55%)
Female	55 (45%)
Race	
Hispanic	2 (1.6%)
Caucasian	101 (83%)
African-American	18 (15%)
Asian	1 (0.8%)
Age (years; mean ± SD)	7.5 ± 4.5
Indication for head MRI	
Evaluation for intracranial malignancy	51 (42%)
Known intracranial malignancy	41 (34%)
Evaluation for pituitary disease	21 (17%)
Evaluation for white matter disease	7 (5.7%)
Trauma	1 (0.8%)
Suspected cerebral vascular accident	1 (0.8%)
Prior chemotherapy	5 (4.1%)
Prior radiation therapy	9 (7.4%)

**Table 2:** Demographic and clinical features of the JIA population. Continuous variables are presented as mean ± sd

Feature	Number
n	35
Sex	
Male	11 (31%)
Female	24 (69%)
Race	
Biracial	1 (2.9%)
Caucasian	28 (80%)
African-American	6 (17%)
Age at MRI (years; mean ± SD)	9.6 ± 4.7
Time from diagnosis to MRI (months; mean ± SD)	2.2 ± 1.8
Time from initial symptom to MRI (months; mean ± SD)	12 ± 17
JIA category	
Oligoarticular	5 (14%)
Systemic	3 (8.6%)
RF- polyarticular	14 (40%)
RF+ polyarticular	3 (8.6%)
Psoriatic	7 (20%)
Enthesitis-related arthritis	3 (8.6%)
Disease activity assessments	
Swollen joint count	8.2 ± 8.3
ESR (*n* = 32; mm / hr)	32 ± 28
Physician global assessment (0–10)	3.3 ± 1.4
Exam findings of TMJ activity or damage	
Maximal incisal opening (*n* = 34; mm)	4.5 ± 0.9
Jaw deviation (*n* = 29)	7 (24%)
Medications	
None	4 (11%)
Prednisone alone	2 (5.7%)
Methotrexate alone	13 (37%)
Biologic alone	3 (8.6%)
Methotrexate plus biologic	13 (37%)

*Abbreviations: ESR* erythrocyte sedimentation rate, *TMJ* temporomandibular joint

### MRI findings (Table 3)

Among the controls, 62 (51%) had an effusion in the TMJ; when present, the mean (± SD) diameter was 0.90 ± 0.22 mm. One-hundred-and-twenty (98%) had contrast enhancement; when present, the mean (± SD) diameter was 1.06 ± 0.24 mm. The maximum sizes of the effusions and enhancement were 1.4 and 1.8 mm, respectively. An illustration of a control subject with abnormal MRI findings is shown in Fig. 1. Among JIA patients, only 10/35 (29%) had an effusion, a statistically significant difference (*p* = 0.022) compared to controls. When present, the sizes of the effusions were non-significantly larger in the JIA patients compared to controls (1.5 ± 1.3 versus 0.89 ± 0.23 mm, *p* = 0.192), and effusions larger than 1.5 mm were only seen in JIA patients (*n* = 3); in contrast, the sizes of the enhancement were statistically significantly larger in the controls compared to patients (1.1 ± 0.24 versus 0.88 ± 0.27 mm, *p* <  0.001). The maximum sizes of the effusions in the controls and JIA patients were 1.4 and 4.5 mm respectively; and for enhancements, the maximum sizes were 1.8 mm in both groups. Chronic changes were seen in none of the controls and in two (5.7%) of the JIA patients (*p* = 0.049). One child had condylar flattening, erosive changes, and pannus; and the other had condylar flattening, osteophytes, and pannus. Both 1.5 T and 3 T MRI scanners were used during the course of the study. The MRI field strength was available on 152 / 157 of the subjects; the 3 T device was used in 24 / 117 (21%) of the control subjects for whom this information was obtainable, compared to 15 / 35 (43%) patients with JIA. Among the controls, the 3 T MRI scanner identified an increased frequency of effusions compared to the 1.5 T MRI (22 / 24 [92%] versus 35 / 93 [38%], respectively, *p* < 0.001). A similar trend was observed among the JIA patients (7 / 15 [47%] versus 3 / 20 [15%], *p* = 0.062). MRI field strength did not appear to impact size of the effusion in either group (data not shown), while 3 T scans were associated with larger sizes of enhancement in controls (1.20 ± 0.20 versus 1.03 ± 0.25 mm, *p* = 0.003) but not patients (data not shown).

**Table 3:** MRI findings in controls compared to JIA patients

Feature	Controls	JIA patients	*p*-value
Frequency of effusion	62 (51%)	10 (29%)	0.022
Size of effusion (mm; mean ± sd)	0.89 ± 0.23	1.5 ± 1.3	0.192
Frequency of enhancement	120 (98%)	34 (97%)	0.533
Size of enhancement (mm; mean ± sd)	1.1 ± 0.24	0.88 ± 0.27	< 0.001
Frequency of chronic changes	0	2 (5.7%)	0.049

To assess the impact of medical therapy on TMJ MRI findings in JIA patients, we tested the correlation of disease duration to the size of effusions and enhancements. While there was no association between effusion size and disease duration from diagnosis to the MRI (*r* = − 0.231, *p* = 0.188), there was a robust inverse association between enhancement and disease duration (*r* = − 0.475, *p* = 0.005). Neither methotrexate nor TNF inhibitors were associated with the presence or size of effusions or contrast enhancement in children with JIA (data not shown), although these findings are likely influenced by the short duration from diagnosis to MRI in many patients.

### Agreement

All of the MRIs of the control subjects were reviewed by both radiologists. When effusions or enhancement were dichotomized to present or absent, agreement between the two radiologists was present in 120 / 122 (98%) for effusion and 122 / 122 (100%) for enhancement. When evaluated on a continuous scale, the two radiologists were within 0.2 mm of each other in 120 / 122 (98%) of subjects with respect to size of the effusion, and in 114 / 122 (93%) subjects with respect to the size of the enhancement. The concordance correlation coefficient indicated inter-reader agreement was 0.98 for effusion and 0.92 for enhancement, representing high levels of agreement for both.

## Discussion

One-hundred-and-twenty-two children were prospectively evaluated for TMJ inflammation while undergoing a clinically indicated MRI of their brains for reasons other than TMJ arthritis screening. Of these subjects, 62 had effusions up to 1.4 mm, and 120 had contrast enhancement up to 1.8 mm. The frequency and size of the areas of effusion and enhancement were similar between JIA patients and controls, with effusions surprisingly more common in the latter. This cannot be explained by MRI field strength, as controls were more likely to have been scanned with a 1.5 T device, which detected a lower frequency of effusions among both groups of subjects. However, the largest effusions were present in those children with JIA, and effusions larger than 1.5 mm were seen only in JIA patients. Thus, our data support several previous studies in this field [9–13], with only the study by Tzaribachev et al. [8] yielding contradictory findings.

The reasons for these widely discrepant findings with respect to the frequency of inflammatory changes in the TMJ of non-arthritic subjects are not entirely clear. To some extent, it may pertain to the study methodology, with respect to looking for specific areas of effusion or enhancement as opposed to dynamic contrast studies that compare the overall pattern of signal intensity pre- versus post-contrast. Additionally, as noted above, retrospective studies by definition involve children who did not undergo dedicated MRI of the TMJ. The field strength of the MRI (1.5 T vs 3 T) may also influence sensitivity with 3 T providing higher resolution images, although the use of TMJ coils in the 1.5 T scanners at least in our institution partially compensated for this limitation. Finally, there may be differences pertaining to the interpretation of the images or the patient populations themselves.

The clear message from this and other recent studies is that small amounts of joint effusion and contrast enhancement are not necessarily pathologic. Perhaps the intensity of the enhancement or the extent of synovial hypertrophy are more appropriate indicators of JIA [10–12]; these were not assessed in our study. It must be emphasized, though, that these findings do not detract from the body of literature indicating that children with JIA are at substantial risk of TMJ arthritis [16]. While the size of the effusions and enhancement in our subjects were typically under 1.5 mm, children with JIA can have changes up to 3–4 mm. Furthermore, none of the 96 children in the study by Tzaribachev [8], only 3 of 46 (6.5%) of the subjects in the study by von Kalle [9], 1 of 27 (3.7%) subjects in the study by Kottke [13], and 0 / 122 in the present study had morphological changes suggestive of advanced TMJ arthritis. In contrast, studies of children with JIA, particularly those conducted prior to the biologic era, demonstrated substantial alterations in the morphology of the TMJ [17, 18]. Such changes can lead to devastating alterations in the form and function of the jaw [17]. Only two JIA subjects in this study had chronic changes by MRI; in part, this is due to the short disease duration, although it is unclear why these results differ from those previously reported in newly diagnosed subjects with JIA, in which chronic changes were seen in 69% at baseline (within 2 months of diagnosis) [5]. It may be related to earlier use of systemic biologic therapies or earlier diagnoses.

We acknowledge some limitations of this study. Although control subjects were screened by questionnaire, no rheumatologist was involved in their evaluation. However, given the rarity of JIA (~ 1 in 1000), it is highly unlikely that any of these subjects were in fact affected with TMJ arthritis. Additionally, some of the control children received chemotherapy or radiation, and many of the JIA subjects were on immunosuppressive therapy, which appeared to have had an effect on the size of the enhancement. There may also have been a delay in the attainment of the images in the control population that was not present in the patients, as the former underwent initial dedicated MRIs of the brain; such a delay could result in increased contrast uptake, thus biasing the study towards findings of increased TMJ inflammation in controls [19]. This is nevertheless important information, as in routine practice, there will likely be variations in the rate at which post-contrast images are obtained, and such information will not necessarily be available to the rheumatologist, underscoring the need to interpret small amounts of contrast uptake with caution. There were also differences in the field strength of the MRI used in patients versus controls, although these differences if anything may have attenuated the differences between the two groups of subjects. Strengths of the study include the following: the number of children evaluated (and in a prospective fashion), the precise measurements of the extent of joint fluid and contrast enhancement, the high level of agreement between the two radiologists, and the comparison with the subjects with JIA.

In conclusion, our study confirms previous findings that a small amount of joint fluid or enhancement can be within the range of normal, particularly when 3 T MRI scanners are used. However, even a small effusion of minimal synovial enhancement may reflect ongoing TMJ arthritis in the setting of a child with JIA. Thus, timely follow-up TMJ MRI examination is necessary to evaluate for progression of disease. This information should be taken into account when interpreting the TMJ MRI of a child with JIA.

## Conclusion

Findings consistent with minimally active TMJ arthritis appear to be equally likely in children with JIA as compared to non-inflamed controls. However, chronic changes and large areas of effusion appear specific for JIA.

## Additional file

Additional file 1: Questionnaire. (JPG 97 kb)

## Abbreviations

CCC: Concordance correlation coefficient
ER: Enhancement ratio
JIA: Juvenile idiopathic arthritis
T Tesla: (MRI field strength)
TMJ: Temporomandibular joint

## References

[1] Krause ML, Crowson CS, Michet CJ, Mason T, Muskardin TW, Matteson EL (2016). Juvenile idiopathic arthritis in Olmsted County, Minnesota, 1960-2013. Arthritis Rheum.

[2] Stoll ML, Sharpe T, Beukelman T, Good J, Young D, Cron RQ (2012). Risk factors for temporomandibular joint arthritis in children with juvenile idiopathic arthritis. J Rheumatol.

[3] Cannizzaro E, Schroeder S, Muller LM, Kellenberger CJ, Saurenmann RK (2011). Temporomandibular joint involvement in children with juvenile idiopathic arthritis. J Rheumatol.

[4] Carmody RN, Gerber GK, Luevano JM (2015). Diet dominates host genotype in shaping the murine gut microbiota. Cell Host Microbe.

[5] Weiss PF, Arabshahi B, Johnson A (2008). High prevalence of temporomandibular joint arthritis at disease onset in children with juvenile idiopathic arthritis, as detected by magnetic resonance imaging but not by ultrasound. Arthritis Rheum.

[6] Muller L, Kellenberger CJ, Cannizzaro E (2009). Early diagnosis of temporomandibular joint involvement in juvenile idiopathic arthritis: a pilot study comparing clinical examination and ultrasound to magnetic resonance imaging. Rheumatology (Oxford).

[7] Lochbuhler N, Saurenmann RK, Muller L, Kellenberger CJ (2015). Magnetic resonance imaging assessment of temporomandibular joint involvement and mandibular growth following corticosteroid injection in juvenile idiopathic arthritis. J Rheumatol.

[8] Tzaribachev N, Fritz J, Horger M (2009). Spectrum Of magnetic resonance imaging appearances of juvenile temporomandibular joints (TMJ) in non-rheumatic children. Acta Radiol.

[9] von Kalle T, Winkler P, Stuber T (2013). Contrast-enhanced MRI of normal temporomandibular joints in children--is there enhancement or not?. Rheumatology (Oxford).

[10] von Kalle T, Stuber T, Winkler P, Maier J, Hospach T (2015). Early detection of temporomandibular joint arthritis in children with juvenile idiopathic arthritis - the role of contrast-enhanced MRI. Pediatr Radiol.

[11] Ma GM, Amirabadi A, Inarejos E (2015). MRI thresholds for discrimination between normal and mild temporomandibular joint involvement in juvenile idiopathic arthritis. Pediatr Rheumatol Online J.

[12] Resnick CM, Vakilian PM, Breen M, et al. Quantifying temporomandibular joint synovitis in children with juvenile idiopathic arthritis. Arthritis Care Res (Hoboken). 2016;69:1795–802. 10.1002/acr.22911 PMC5573997 27110936

[13] Kottke R, Saurenmann RK, Schneider MM, Muller L, Grotzer MA, Kellenberger CJ (2015). Contrast-enhanced MRI of the temporomandibular joint: findings in children without juvenile idiopathic arthritis. Acta Radiol.

[14] Vaid YN, Dunnavant FD, Royal SA, Beukelman T, Stoll ML, Cron RQ (2014). Imaging of the temporomandibular joint in juvenile idiopathic arthritis. Arthritis Care Res (Hoboken).

[15] Lin LI (1989). A concordance correlation coefficient to evaluate reproducibility. Biometrics.

[16] Stoll ML, Cron RQ (2015). Temporomandibular joint arthritis in juvenile idiopathic arthritis: the last frontier. Int J Clin Rheumatol.

[17] Stabrun AE (1991). Impaired mandibular growth and micrognathic development in children with juvenile rheumatoid arthritis. A longitudinal study of lateral cephalographs. Eur J Orthod.

[18] Larheim TA, Hoyeraal HM, Stabrun AE, Haanaes HR (1982). The temporomandibular joint in juvenile rheumatoid arthritis. Radiographic changes related to clinical and laboratory parameters in 100 children. Scand J Rheumatol.

[19] Rieter JF, de Horatio LT, Nusman CM, et al. The many shades of enhancement: timing of post-gadolinium images strongly influences the scoring of juvenile idiopathic arthritis wrist involvement on MRI. Pediatr Radiol. 2016;46:1562–7. 10.1007/s00247-016-3657-0 27406611

