# Supplementary figures and images for: Defining the normal appearance of the temporomandibular joints by magnetic resonance imaging with contrast: a comparative study of children with and without juvenile idiopathic arthritis
Source: Pediatr Rheumatol Online J. 2018 Jan 24;16:8. doi: 10.1186/s12969-018-0223-3 (PMC5784616; doi:10.1186/s12969-018-0223-3)

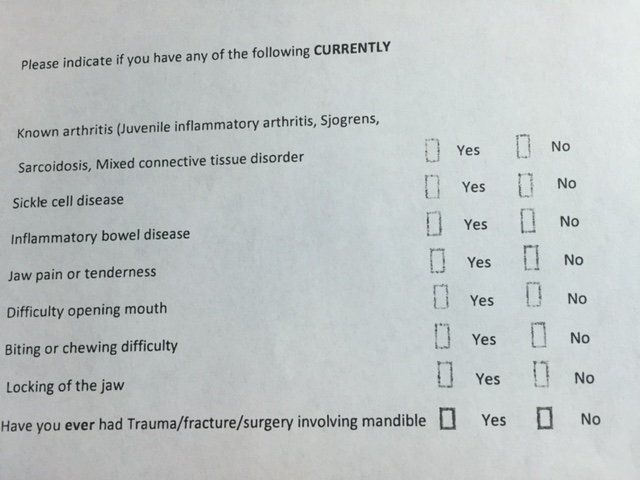

Supplement: Additional file 1: — Questionnaire. (JPG 97 kb) [file 12969_2018_223_MOESM1_ESM.jpg]
